# An absorbed dose‐based source strength determination for diffusing alpha‐emitters radiation therapy (DaRT) brachytherapy seeds – Proof of concept

**DOI:** 10.1002/mp.70167

**Published:** 2025-11-29

**Authors:** Sean P. Jollota, Jeffrey L. Radtke, Larry A. DeWerd, Ahtesham Ullah Khan

**Affiliations:** ^1^ Department of Medical Physics School of Medicine and Public Health University of Wisconsin‐Madison Madison Wisconsin USA

**Keywords:** absorbed dose, alpha‐emitting radionuclides, brachytherapy, dosimetry, ion chamber, Monte Carlo

## Abstract

**Background:**

Diffusing alpha‐emitter radiation therapy (DaRT) is currently being evaluated in several clinical trials as an interstitial temporary brachytherapy source. However, determining source strength solely based on gamma spectrometry measurements of ^224^Ra activity has limitations. This method does not account for changes in the desorption of ^224^Ra progeny from the seed's surface or the therapeutically relevant alpha emissions. Variations in the source construction, especially ^224^Ra depth in the seed, significantly impact the ^224^Ra progeny desorption probabilities which leads to changes in the dose deposited in the tumor. Therefore, an improved source strength specification for DaRT seeds is needed, one that accounts for variations in source construction that affects the absorbed dose delivered to the tumor.

**Purpose:**

There were two aim of this work. The first aim was to investigate the impact of ^224^Ra distribution depth on progeny desorption probabilities and correspondingly absorbed dose to water using both Monte Carlo (MC) simulations and the diffusion‐leakage (DL) model. The second aim was to propose an absorbed dose‐based source strength specification and assess the sensitivity of the proposed standard to variations in the desorption probabilities.

**Methods:**

A MC model of the DaRT seed was constructed with varying ^224^Ra distribution depths ranging from 0 to 14 nm. The corresponding ^220^Rn and ^212^ Pb desorption probabilities were calculated using alpha and gamma emissions originating inside the source, respectively. The calculated desorption probabilities were utilized as input parameters to the DL model, which was solved using a finite element analyzer, in order to calculate the cumulative absorbed dose to tumor over the treatment period. The design considerations for the proposed absorbed dose standard were outlined along with a MC study of the expected signal from a 3 µCi DaRT seed. The sensitivity of the proposed source strength to varying desorption probabilities was assessed.

**Results:**

The progeny desorption was found to be highly sensitive to the distribution depth parameter with a sharp reduction in desorption probabilities with increasing distribution depth. The cumulative absorbed dose to tumor was found to vary drastically with the desorption probabilities with dose differences of up to 80%. Despite large dose differences, the 10‐Gy prescription isodose line was found to be within 1 mm of each other indicating a spatial shift due to differences in desorption. The absorbed dose standard was found to have a signal > 7 pA with high sensitivity to the changes in the desorption probabilities. The MC‐calculated correction factors were found to be < 3% with negligible dependence on the changes in desorption.

**Conclusions:**

This work investigated and reported a large dependence of absorbed dose to water on desorption of ^224^Ra progeny. An absorbed dose‐based source strength specification was proposed with a preliminary design of the apparatus. Using MC methods, the proposed instrument was deemed suitable for source strength measurements with a high sensitivity to changes in the DaRT seed construction.

## 1 INTRODUCTION

Diffusing alpha‐emitter radiation therapy (DaRT) is a brachytherapy technique, where ^224^Ra radionuclide is plated on a stainless steel wire, heated, coated with a polymer, and inserted interstitially for brachytherapy treatment of cancer.^1^ The initial concern of using an alpha‐emitting radionuclide for brachytherapy applications was ensuring the progeny radionuclides and corresponding alpha‐emissions could travel several millimeters of tissue to deposit a therapeutic dose to the tumor.^2^, ^3^, ^4^ Due to this short range, the DaRT sources’ dose distribution is dependent on progeny radionuclides' diffusive transport within the tumor.^5^ Initial findings for the DaRT source demonstrated therapeutic dose distributions extending up to approximately 5–6 mm from the wire in tissue^6^, ^7^, ^8^, ^9^, ^10^ which has been supported by more recent studies recommending a clinical seed spacing of 5 mm in hexagonal configurations.^11^, ^12^ Initially in the U.S., the results of the first human pilot study involving 10 patients with keratinocyte carcinomas were reported as a 100% complete response, although long‐term outcomes for this cohort are still under evaluation.^13^ Other clinical trials have also demonstrated promising results using the DaRT sources.^14^, ^15^, ^16^, ^17^ Currently, according to the Alpha Tau Medical website, there are five active clinical trials for skin cancer, three for oral cancer, one for prostate cancer, two for pancreatic cancer, one for breast cancer, one for liver cancer, and one for lung cancer internationally.^18^

The activity of DaRT sources is measured before treatment and converted to absorbed dose distribution using the diffusion leakage (DL) model.^19^ Initially, the DL model approximated the source as a point source and served to calculate the dose distribution from the radionuclides in the ^224^Ra decay chain by accounting for the diffusive transport of the progeny intratumor.^19^ This model was later adapted to account for a 2D geometry but only accounted for the alpha emissions of the decay chain.^11^, ^12^ A new adaptation of this model has been combined with dose point kernels (DPKs) of the beta decays in the ^224^Ra chain to calculate the total dose distribution.^20^ The DL model is primarily utilized to determine the inter‐seed spacing for a given treatment volume.

The dose distribution inside the tumor from a DaRT seed is dependent on several parameters, including the ^224^Ra activity, progeny desorption probabilities, uniformity of ^224^Ra plating on the seed, effective diffusion length of progeny in the treatment medium (influenced by tissue type, vascularity, and immune response), and clearance of activity from the tumor.^19^ The ^224^Ra activity and progeny desorption probabilities are primarily determined by source construction, which may vary between batches or even individual seeds.^11^ Differences in seed preparation methods, such as singly heat‐treated seeds versus seeds additionally coated with a polymer layer, and the behavior of specific progeny isotopes such as ^220^Rn and ^212^ Pb, further impact the resulting dose distribution.^19^

Following the electrostatic attraction of ^224^Ra onto the surface of the seed, the seed is heated to promote migration of ^224^Ra to a shallow depth below the surface, ensuring its retention within the wire while still allowing the escape of recoiling alpha‐emitting progeny radionuclides.^19^ Due to the source's construction, alpha particles emitted by ^224^Ra and its progeny can deposit dose to tissue from two primary origins: (1) desorbed progeny, where alpha particles interact with tissue without attenuation, and (2) decays occurring within the source itself, including those within the wire and polymer coating.^21^

The electrostatic attraction of ^224^Ra onto the stainless steel surface inherently causes a nonuniform distribution, both between different seeds and within individual seeds. This non‐uniformity can influence progeny desorption due to local variations in distribution depth and surface coverage. Physical validation of this depth by the manufacturer is accomplished by ensuring that no ^224^Ra escapes from the seed.^2^ Thus, the desorption probabilities are dependent on the distribution depth parameter.^22^ Although previous studies have investigated the impact of ^224^Ra activity, clearance probability, and diffusion lengths on intratumoral dose distribution, the role of progeny desorption probabilities from DaRT seeds remains underexplored.^12^ Notably, Fedorchenko et al. discussed desorption in the context of ^224^Ra distribution depth and material interfaces; however, their work focused primarily on estimating escape probabilities at specific locations rather than modeling the downstream effects of desorption on spatial dose distributions.^23^ This study builds upon those insights by explicitly incorporating variable desorption probabilities into a dosimetric model to assess their impact across tumor volumes. This work utilizes Monte Carlo (MC) methods to investigate the relationship between the ^224^Ra distribution depth and progeny desorption probabilities with the emphasis on understanding the changes in absorbed dose to tumor over the treatment duration.

AAPM Task Group 56 recommends that calibrations of all brachytherapy sources be directly traceable to the National Institute of Standards and Technology (NIST).^24^ However, such calibrations currently do not exist for the DaRT seeds. The source strength for an individual DaRT seed is currently dictated by the ^224^Ra activity, measured in disintegrations per second. However, activity is not directly correlated with the energy emitted from the DaRT source or the dose absorbed in the tumor, which can vary due to differences in source preparation techniques, polymer coating attenuation, and the homogeneity of ^224^Ra on the wire. The polymer coating serves three main purposes: (1) it protects the ^224^Ra layer during handling and implantation, (2) it enhances biocompatibility in tissue, and (3) it prevents recoiling ^224^Ra atoms from escaping into surrounding tissue.^21^ However, the polymer also attenuates alpha emissions, affecting the dose delivered to the tumor. This highlights that gamma‐based measurements of ^224^Ra activity do not fully account for the actual dose delivered to tissue, as they neglect factors such as progeny desorption and alpha attenuation. To achieve precise dose delivery and minimize the risk of dose heterogeneity, it is crucial that the source strength specification reflects the absorbed dose delivered to the tumor and has high sensitivity to changes in source construction.

Absorbed dose measurements from alpha particles are challenging due to their short range, which is highly energy‐dependent. For the alpha particles emitted from ^224^Ra and its progeny, energies range from approximately 5–9 MeV, corresponding to penetration depths of less than 100 µm in water or tissue.^4^ This limited range makes conventional thick‐walled ionization chambers unsuitable for direct measurement of alpha emissions. However, thin‐window parallel‐plate ionization chambers, with sufficiently low‐density entrance windows, are well‐suited for this task. Ionization chambers have been used as primary standards for absorbed dose for beta‐emitting sources used in brachytherapy applications.^25^, ^26^, ^27^ More recently, the use of ionization chambers has expanded to measurements of solution‐based sources, beta‐emitting ophthalmic applicators, and ^210^Po, an alpha‐emitting radionuclide.^28^, ^29^, ^30^, ^31^ This work proposes an absorbed dose formalism for the DaRT source using a thin‐window parallel‐plate ionization chamber. Prior to building the actual apparatus, it is pertinent that the design of the device meets all specifications required for absorbed dose measurements from DaRT seeds.

Therefore, the aim of this proof‐of‐concept study was to utilize MC modeling guidance to design the absorbed dose standard and to assess the expected signal for the proposed measurement apparatus and the relevant correction factors. Once an absorbed dose standard has been devised for DaRT seeds, any changes in the source strength and construction will reflect in the absorbed dose measurements allowing an adjustment of source strength or seed placement pattern to match the prescribed absorbed dose to tumor.

## 2 MATERIALS AND METHODS

### 2.1 The DaRT source

**FIGURE 1:**
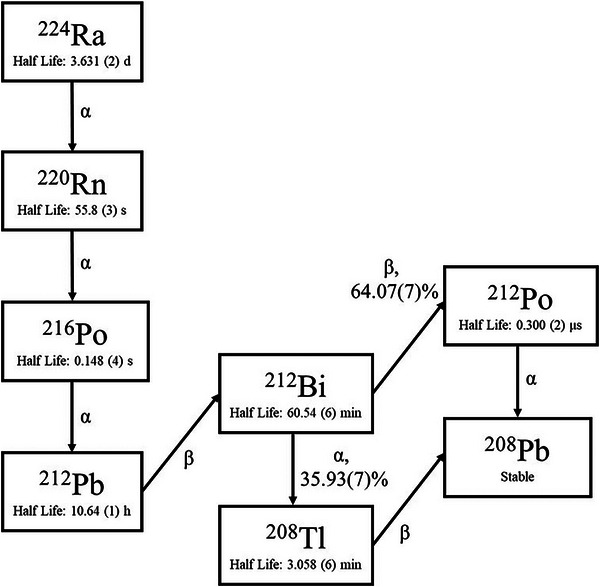
^224^Ra decay chain with decay emissions, half‐lives, and branching ratios taken from Decay Data Evaluation Project (DDEP).^3^ ^.^

The DaRT source geometry used in this study is based on the specifications reported by Popovtzer et al., where ^224^Ra is plated onto a hollow stainless steel wire with an outer diameter of 0.7 mm and a length of 1.0 cm.^14^ For clinical applications, the DaRT source is typically loaded with an activity of 3 µCi at the time of treatment.^32^ In this study, a 30‐day treatment period was assumed to reflect the clinically relevant duration over which the dose is delivered following DaRT source implantation. The ^224^Ra decay chain comprises of five possible alpha decays and three possible beta decays, ultimately resulting in stable ^208^ Pb, shown in Figure 1. Following the initial decay of ^224^Ra, an alpha emission produces ^220^Rn, a noble gas capable of diffusing away from the seed surface and through surrounding tissue.^2^ This mobility introduces a mechanism of activity clearance from the tumor, which is influenced more by tissue compactness than vascularity, as diffusion is more restricted in dense tissue like muscle compared to less compact tumor tissues. Recent work by Hinrich et al. has further explored the solubility and permeability of ^220^Rn in various tissues, while Heger et al. have identified ^220^Rn as the primary progeny influencing diffusion from the DaRT source.^33^, ^34^

In addition to ^220^Rn, ^212^ Pb also contributes significantly to progeny transport within tissue. While ^220^Rn is the dominant diffusing progeny following ^224^Ra decay in air, ^212^ Pb is primarily redistributed in tissue through nuclear recoil implantation and subsequent biological processes. Due to its longer half‐life (10.6 h), ^212^ Pb is capable of migrating further from the decay site before subsequent decay events occur. This dual progeny mobility, particularly of ^220^Rn and ^212^ Pb, is a critical aspect of the decay chain that influences the spatial distribution of dose within the tumor. These mobile species contribute to both localized dose deposition and activity clearance, which can affect the overall absorbed dose profile. ^224^Ra reaches radioactive equilibrium with its progeny approximately six days after separation, as determined by the solution of the Bateman equations.^35^ This equilibrium timing is driven by the half‐life of ^212^ Pb (10.6 h) relative to ^224^Ra (3.6 days).

### 2.2 Impact of desorption on absorbed dose

The Tool for Particle Simulation (TOPAS) MC code Version 3.7, which is a GEANT4 wrapper created for heavy‐charged particle simulations, was used for all simulations done in this work.^36^ The simulation parameters are compiled in Table 1, in accordance with the AAPM TG‐268 report.^37^ A custom electromagnetic physics list was used in this work, which was previously validated for alpha particle transport algorithm.^38^ The desorption probability of the ^224^Ra progeny is highly dependent on accurate modeling of the Coulomb scattering process for the progeny charged particles. Therefore, single Coulomb scattering process was turned on for all ions and alpha particles along with a maximum step size of 0.1 nm. A combination of six other physics lists was used in this work: “g4h‐phy_QGSP_BIC_HP”, “g4decay”, “g4ion‐binarycascade”, “g4h‐elastic_HP”, “g4stopping”, and “g4radioactivedecay”. The production threshold for all particles was set to 0.1 nm. In the MC simulations, the DaRT source was modeled as a hollow stainless steel wire composed of 316 LVM stainless steel, with an outer diameter of 0.7 mm and a length of 1.0 cm, consistent with the geometry described in Popovtzer et al.^14^ In this study, the polymer coating was not simulated, as the primary objective was to isolate the effects of progeny desorption and its contribution to the absorbed dose. Future work may incorporate the polymer layer to evaluate its impact on dose attenuation and recoil containment.

**TABLE 1:** Monte Carlo (MC) simulation parameters used in this work.

Item name	Description
Code version	TOPAS v3.7
Validation	Khan and DeWerd^38^, Arce et al.^39^, Fedorchenko et al.^23^
Timing	∼1 h
Transport parameters	Modified G4EMStandardOpt4 parameters^38^ and using Single Scattering model for ions and alpha particles^23^
Variance reduction	None
Scored quantity	Absorbed dose to water
Statistical uncertainty	One million histories were run to achieve uncertainty < 1%
Postprocessing	None
	

The 10 × 10 × 10 cm^3^ volume surrounding the simulated seed was set to vacuum to ensure that desorbed progeny radionuclides escape the simulation without decaying. The ^224^Ra radionuclide was simulated at depths of 0.0–14.0 nm, consistent with depths reported by Alpha Tau Medical for DaRT seed fabrication, to study the impact of distribution depth on progeny desorption probabilities. A phase space file was scored to extract alpha and gamma spectra for each ^224^Ra distribution depth in order to calculate the desorption probabilities:^19^

(1) $$P_{des}\left(Rn\right)=1-\frac{A_{Rn}^{\alpha }}{A_{Ra}^{\alpha }}$$

(2) $$P_{des}\left(Pb\right)=1-\frac{A_{Pb}^{\gamma }}{A_{Ra}^{\gamma }}$$

where $P_{des}\left(Rn\right)$ is the desorption probability of ^220^Rn and $P_{des}\left(Pb\right)$ is the desorption probability of ^212^ Pb. The activities, $A_{Ra,Rn,Pb}^{\alpha ,\gamma }$, were determined in MC using Equation (3) and are expressed in units of becquerels (Bq):

(3) $$A_{Ra,Rn,Pb}^{\alpha ,\gamma }=\frac{C_{Ra,Rn,Pb}^{\alpha ,\gamma }}{I_{Ra,Rbnn,Pb}^{\alpha ,\gamma }}$$

where $C_{Ra,Rn,Pb}^{\alpha ,\gamma }$ is the counts from the phase space file for the alpha peaks of ^224^Ra (5.6855 MeV) and ^220^Rn (6.288 MeV) and the gamma peaks of ^224^Ra (0.240 MeV) and ^212^ Pb (0.239 MeV). $I_{Ra,Rbnn,Pb}^{\alpha ,\gamma }$ represents the known emission intensities for the alpha emissions from ^224^Ra (94.73%) and ^220^Rn (99.88%) and the gamma emissions from ^224^Ra (4.12%) and ^212^ Pb (43.6%).^3^ It is of note that the phase space file was filtered to ensure that only alpha and gamma particles emitted from within the DaRT seed were scored, rather than those emitted from progeny radionuclides that had desorbed from the seed surface. This approach isolates the contribution of radiations from the source itself.

A finite element analyzer (COMSOL Multiphysics) was employed to solve the DL model in a cylindrical coordinate system, as described in our previous work.^20^ The initial ^224^Ra activity was set to 3 µCi with the ^212^ Pb leakage probability of 0.5, consistent with simulation parameters reported in previous work.^12^ The DL model was solved independently for the corresponding $P_{des}\left(Rn\right)$ and $P_{des}\left(Pb\right)$ extracted for each ^224^Ra distribution depth parameter ranging from 0 to 14 nm. Based on the in‐vivo experimental data reported by Heger et al., the ^220^Rn diffusion length was assigned to 0.4 mm.^40^ Both low diffusion and high diffusion cases were investigated with the ^212^ Pb diffusion lengths set to 0.1 and 0.6 mm, respectively.^19^ All other parameters remained unchanged from our previous work.^20^ It is important to note that the DL COMSOL model was not applied to the ionization chamber measurements. This model was used solely for the initial investigation to assess the impact of progeny desorption on absorbed dose in tissue‐like environments. Absorbed dose profiles along the radial direction were reported at the mid‐plane (center) of the z‐axis of the DaRT source geometry.

### 2.3 Absorbed dose‐based source strength specification

Direct measurement of absorbed dose from alpha‐emitting radionuclides using ionization chambers is challenging.^28^ The two major challenges associated with measuring absorbed dose for DaRT seeds include alpha attenuation and the desorbed progeny radionuclides, especially ^220^Rn, contaminating the apparatus. Placing the DaRT seed in a media with high density will significantly attenuate the alpha particles limiting measurements of absorbed dose. In‐air placement of a DaRT seed would reduce alpha attenuation. However, such an experimental setup entails that at a given spatial location, radiation fluence would include emissions directly from the radionuclides decaying inside the source as well as from desorbed ^220^Rn and recoil‐implanted ^212^ Pb progeny that may migrate away from the seed due to electrostatic fields or air movement. While ^212^ Pb does not exhibit true gaseous diffusion, its migration through charge‐assisted transport or convection could still contribute to contamination of the measurement apparatus. Thus, the signal for this setup at a given time will be dependent on the location of the desorbed progeny and requires handling of ^220^Rn gas inside the apparatus. Such an experimental setup complicates dosimetry with added radiation safety concerns. Therefore, we propose an apparatus akin to the absorbed dose standard proposed in our previous work with a few modifications.^31^

**FIGURE 2:**
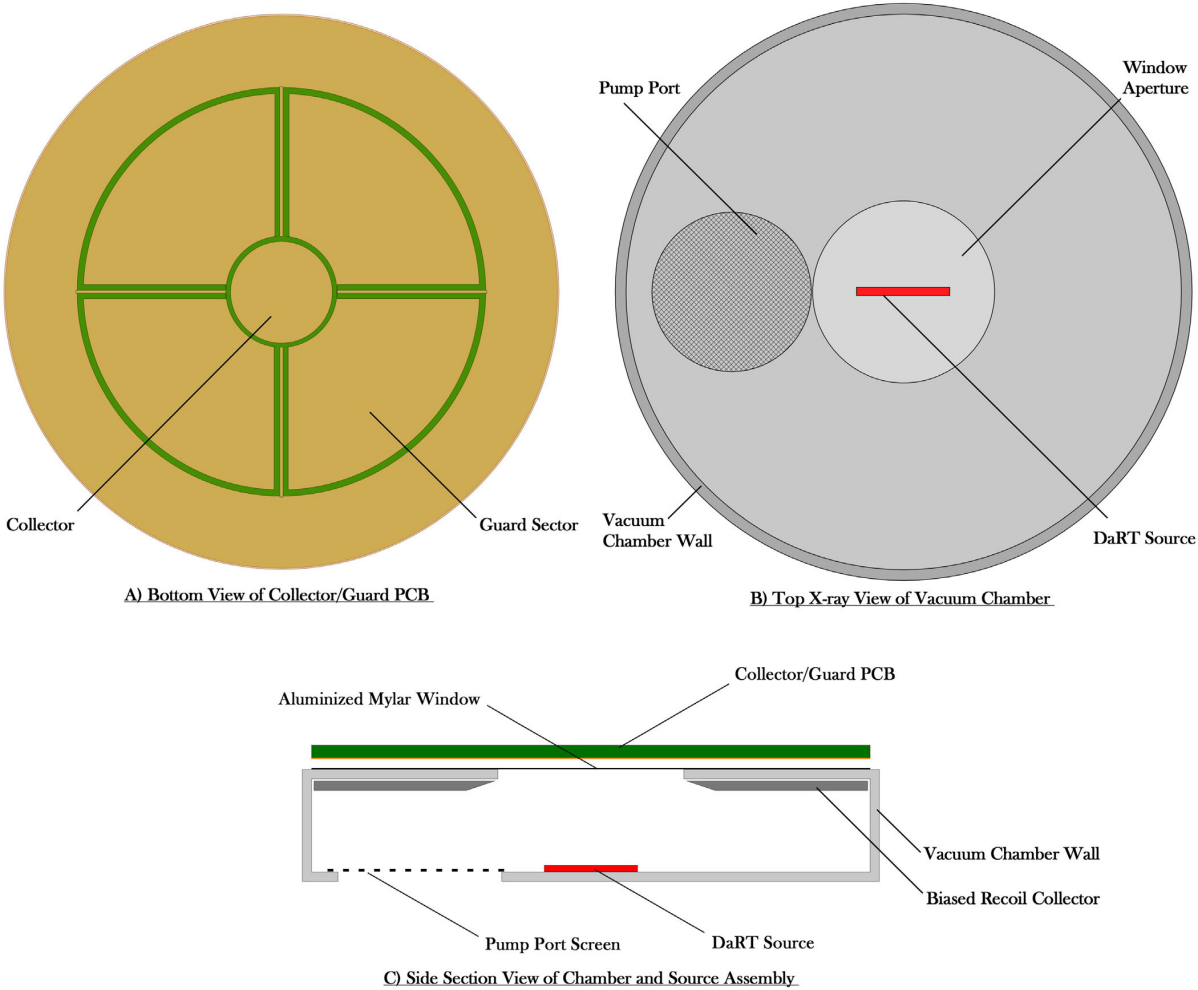
Schematic of the proposed vessel to measure absorbed dose from a DaRT seed.

Figure 2 illustrates the multi‐view schematic of the apparatus in three perspectives: (a) the bottom view of the collector/guard printed circuit board (PCB), highlighting its segmented configuration for optimized lateral and parallel alignment; (b) the top x‐ray view of the vacuum chamber, depicting the lateral placement of the pump port, the DaRT source centered in the chamber, and the window aperture designed for emission collimation; and (c) the side section view of the chamber and source assembly, demonstrating the aluminized Mylar window positioned above the DaRT source, and the pump port screen for vacuum flow. To suppress detection of recoil progeny, the system includes a biased recoil collector, which prevents attachment of the desorbed, charged radionuclides to surfaces within alpha range of the aluminized mylar window. As illustrated in Figure 2c, the biased recoil collector is positioned near the DaRT source to capture desorbed progeny that are not ^220^Rn, ensuring that only the intended emissions are measured in the parallel‐plate ionization chamber. In addition, the vacuum environment is designed to continuously evacuate ^220^Rn from the chamber, minimizing its residence time and preventing surface deposition. The effectiveness of the recoil collector is strongly dependent on the extent of ^220^Rn clearance and the fraction of non‐^220^Rn progeny emitted via recoil from the DaRT surface, both of which are highly variable and better suited for experimental measurement than simulation.

The DaRT seed will reside inside a low‐pressure vessel, continuously pumped to effectively remove the desorbed progeny radionuclides from the apparatus, minimizing radiation safety concerns, desorbed progeny signal, and alpha attenuation. A large (2 cm) diameter pump port will ensure viscous flow conditions, for efficient pumping. A circular hole in the vessel will accommodate a thin (3 µm) aluminized mylar window to collimate the radiation fluence allowing particles to escape the vessel while minimizing alpha attenuation. A small (3 L/s) rough‐vacuum pump will reduce the average progeny residence time in the 6 cm^3^ vessel to 2 ms. So, the radiation fluence incident on the detector will be entirely from the particles emitted from inside the surface of the source. The DaRT seed will be placed on a groove such that the mylar window is 1 cm away from the seed. A 1 cm source‐to‐window distance was selected to balance maximizing the collected solid angle and achieving sufficient signal‐to‐noise ratio (SNR), without introducing excessive particle attenuation or angular spread. Outside the vessel facing the mylar window, a windowless parallel‐plate IC will be placed constructed out of a PCB, as described in our previous study.^31^ The use of a thin film of aluminized mylar as the entrance window establishes the electric field and defines the sensitive volume. To maximize SNR and collect particles from the entire length of the DaRT seed, integral absorbed dose was chosen to be measured with the air cavity diameter (2 cm) being larger than the DaRT seed length (1 cm) and the cavity thickness of 1 mm. With these parameters, we hypothesize that the proposed absorbed dose standard will retain high SNR, minimal correction factors, and high sensitivity to changes in the DaRT source construction such as ^224^Ra activity and desorption probabilities.

The proposed source strength specification is integral absorbed dose‐to‐air at 1 cm distance away from the source measured in‐vacuo. Radioactive equilibrium between ^224^Ra and its progeny was assumed. Absorbed dose, in Gy/s, can be calculated as:

(4) $$\begin{array}{cc} \overset{.}{\underset{air}{D}} & =\frac{k_{MC,\alpha }{\left(\frac{\bar{W}}{e}\right)}_{air,\alpha }+k_{MC,\beta }{\left(\frac{\bar{W}}{e}\right)}_{air,\beta }}{\rho_{0}\cdot \pi r^{2}}\cdot \frac{I}{l}\\ & \;\times \cdot \left(k_{tp}k_{ion}k_{pol}k_{elec}\right)\cdot \left(k_{window}k_{scatter}\right) \end{array}$$

where ${\left(\frac{\bar{W}}{e}\right)}_{air}$ is the mean energy required to create an ion pair in air. For alpha particles, this has been previously determined to be 34.96 J/C with an uncertainty of 0.2%.^41^, ^42^ For electrons, the recommended value given by ICRU 90 is 33.97 J/C with a relative standard uncertainty of 0.15%.^43^ A MC weighting factor, $k_{MC,\alpha }$ or $k_{MC,\beta }$, based on dose contributions from alpha‐emissions and beta‐emissions was applied to ${\left(\frac{\bar{W}}{e}\right)}_{air}$. $\rho_{0}$ is the density of air at standard temperature and pressure, in kg/m^3^. $\pi r^{2}$ accounts for the sensitive measurement area of the ion chamber, with r expressed in meters. I is the measured ionization current, in amperes, and l is the air gap distance between the entrance window and the collecting electrode, in meters. A capacitance‐based measurement of the effective air gap and parallelism between the window and the collector will be performed.^28^ The initial series of correction factors, $k_{tp}k_{ion}k_{pol}k_{elec}$, are considered to be measurement‐based correction factors.^28^ The second series of correction factors, $k_{window}k_{scatter}$, are the MC‐based correction factors. These factors are one of the main drivers in determining an optimal geometry for the measurement of absorbed dose to air for the DaRT source. $k_{window}$ correction factor accounts for the presence of the mylar window and is calculated as the ratio of dose to air cavity without and with the presence of the mylar window. A thin window is also necessary for the measurement of the alpha particles in the plateau portion of their path instead of closer to their Bragg peak, where the current readout would be more sensitive to positional uncertainty. $k_{scatter}$ correction accounts for the in‐air scatter near the air cavity and for the presence of the IC components and the vessel walls. This correction is dependent on the determination of material type for the collecting electrode and the vessel. It has been reported that copper versus tissue equivalent material for the collecting electrode does not affect alpha particle backscatter but can affect backscatter for electrons.^44^ So, the magnitude of this effect was quantified.

The DaRT seed MC model from Section 2.2 was employed and the proposed apparatus was simulated in the TOPAS code. The low‐pressure vessel wall was simulated as 1 mm thick composed of steel. Phase space files scored at the surface of the seed were used for all simulations. The desorbed progeny radionuclides were discarded mimicking the experimental setup. A PCB‐based IC with copper electrodes was simulated and placed against the mylar window low pressure vessel. Absorbed dose rate for a 3 µCi DaRT seed was simulated. A determination of the signal output from these simulations was calculated using the following equation:

(5) $$I=\overset{.}{\underset{air}{D}}\cdot \frac{\rho_{0}\cdot \pi r^{2}\cdot l}{k_{MC,\alpha }{\left(\frac{\bar{W}}{e}\right)}_{air,\alpha }+k_{MC,\beta }{\left(\frac{\bar{W}}{e}\right)}_{air,\beta }}\cdot A_{0}$$

$\overset{.}{\underset{air}{D}}$ is in‐air dose rate, $A_{0}$ is the ^224^Ra activity of the DaRT source, which is given as 3 µCi at the time of patient insertion, r is the radius of air cavity set to 1 cm, l is the thickness of the air cavity simulated as 1 mm. The parameters $k_{MC,\alpha }$ and $k_{MC,\beta }$ are MC–derived dose‐weighted factors representing the fraction of the total absorbed dose contributed by alpha and beta emissions, respectively. Separate dose contributions were isolated using MC scoring filters applied to alpha and beta particles during the simulation. These simulations were repeated for desorption probabilities corresponding to the ^224^Ra distribution depths of 0 to 1 nm with 0.2 nm increments. The $k_{window}$ and $k_{scatter}$ correction factors were computed as a function of ^224^Ra distribution depth to ensure that these corrections remain consistent with the change in desorption probabilities.

## 3 RESULTS

### 3.1 Impact of desorption on absorbed dose

**FIGURE 3:**
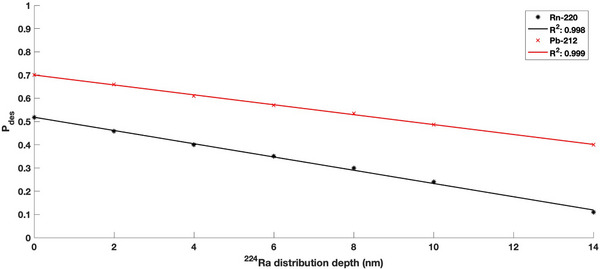
The ^220^Rn and ^212^ Pb desorption probabilities as a function of the distribution depth parameter.

The MC‐calculated ^220^Rn and ^212^ Pb desorption probabilities are displayed in Figure 3. With the increase in ^224^Ra distribution depth, the desorption of progeny radionuclides decreases drastically due to the lack of required recoil kinetic energy to escape the DaRT seed. The ^220^Rn desorption probability was found to decrease from 0.51 to 0.11 with the increasing ^224^Ra distribution depth displaying a strong dependence on the distribution depth parameter. The ^212^ Pb desorption probability was noted to be higher than the ^220^Rn values with a decrease from 0.7 to 0.4 with the increase in ^224^Ra distribution depth.

**FIGURE 4:**
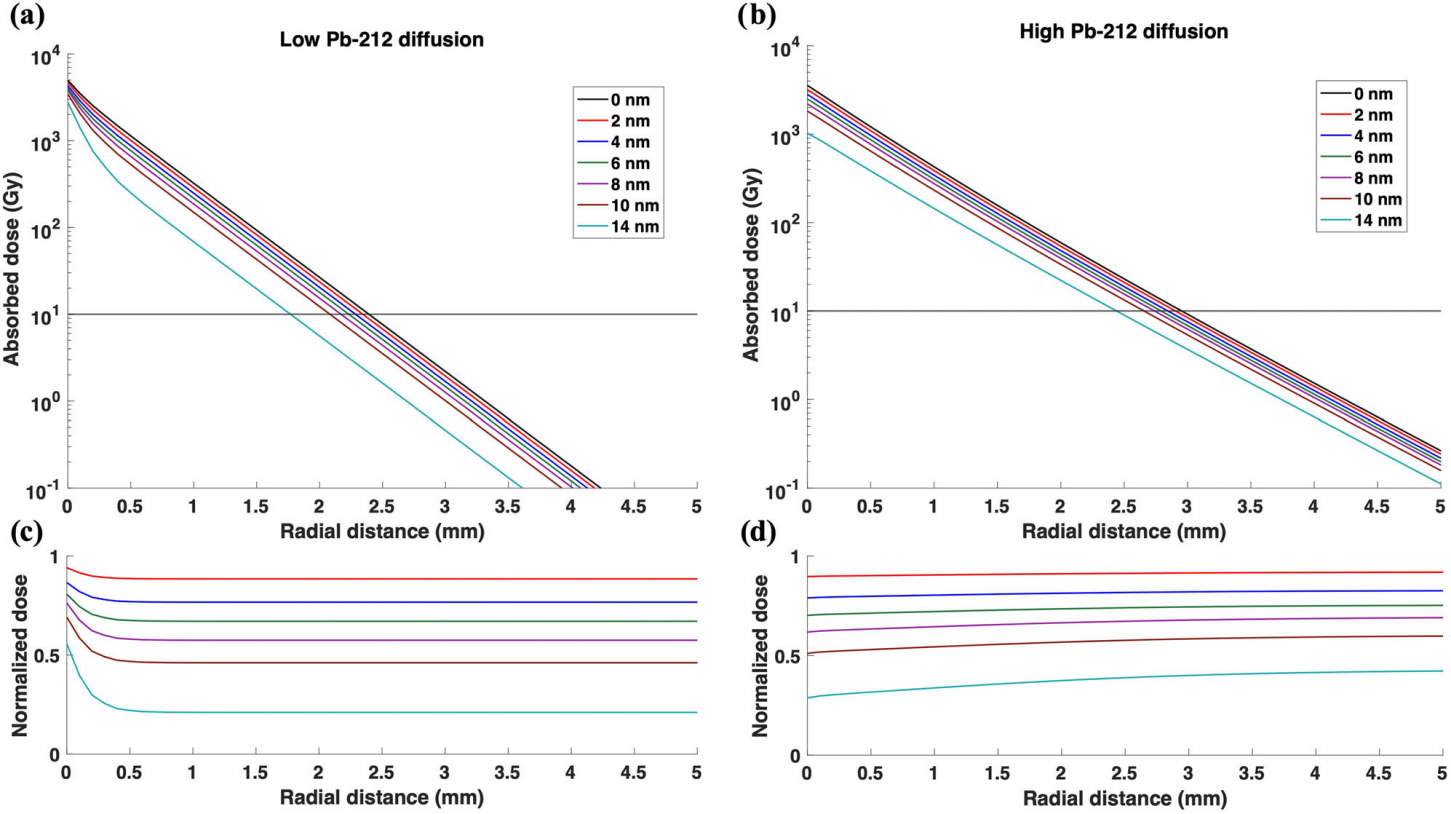
The cumulative absorbed dose to water radial profiles for a 30‐day treatment period for both (a) low ^212^ Pb diffusion case (diffusion length = 0.1 mm) and (b) high ^212^ Pb diffusion case (diffusion length = 0.6 mm) for various ^220^Rn and ^212^ Pb desorption probabilities corresponding to the investigated range of ^224^Ra distribution depth parameter. Dose profiles normalized to the profile for the 0 nm distribution depth are also displayed for both (c) low diffusion and (d) high diffusion cases. The same legend applies to all subfigures. The horizontal line represents the 10 Gy prescription dose.

Figure 4 reports the total absorbed dose to water over the treatment period for the calculated desorption probabilities shown in Figure 3. A leakage probability of 0.5 for ^212^ Pb, consistent with previous work, was incorporated into the dose calculations to account for progeny migration effects.^20^ Both low diffusion and high diffusion cases were considered. As expected, the decrease in desorption probabilities was found to be correlated with the decrease in absorbed dose. Additionally, a strong dependence on desorption was reported with dose differences of up to 80% due to a high dose gradient. For the high diffusion case, a consistent shift in the radial distance to 10 Gy prescription dose was noted. The distance to 10 Gy prescription dose decreased from 3.0 to 2.4 mm with the increase in ^224^Ra distribution depth. A relatively consistent shift was noted with every 2 nm increase in distribution depth for the high diffusion case. The overall shift in the 10 Gy prescription isodose line was observed to be similar for the low diffusion case with a decrease from 2.4 to 1.7 mm radial distance. However, this shift of the 10 Gy isodose line becomes more pronounced as the distribution depth increases for the low diffusion case due to the ^212^ Pb‐dominated dose distribution limiting the spread of alpha particles away from the source.

### 3.2 Absorbed dose‐based source strength specification

**FIGURE 5:**
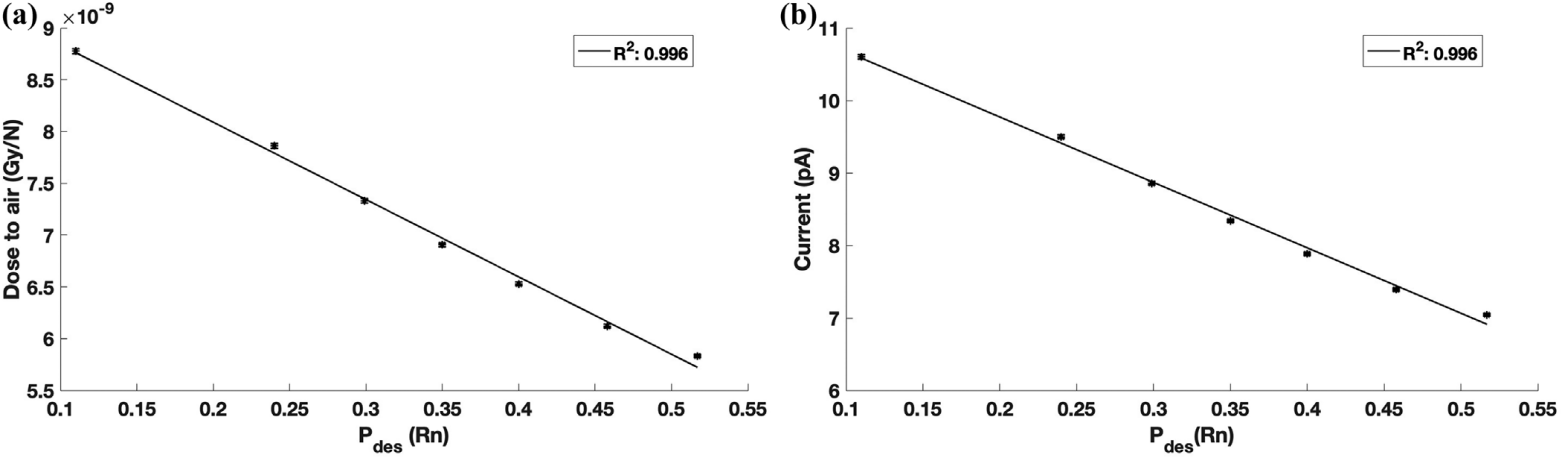
(a) The calculated absorbed dose to air per unit disintegration of ^224^Ra as a function of ^220^Rn desorption probability ($P_{des}\left(Rn\right)$). (b) The estimated detector signal for a 3 µCi ^224^Ra source for various $P_{des}\left(Rn\right)$. The error bars correspond to 1σ uncertainty and are smaller than the markers.

The MC‐calculated absorbed dose to air cavity normalized to a single ^224^Ra disintegration is plotted in Figure 5a. As desorption increases, the dose deposited in the air cavity decreases since the progeny radionuclides are removed from the apparatus shown in Figure 2 leading to a reduction in radiation fluence incident on the detector. The absorbed dose contributions from alpha particles were found to be over 80% with the remaining absorbed dose deposited by the delta‐rays produced by the alpha particle tracks. The beta particle contribution to the total absorbed dose was calculated to be < 1% across the investigated distribution depths. Therefore, the majority of the signal is generated by the incident alpha particles. For a 3 µCi ^224^Ra DaRT seed, the estimated detector signal is shown in Figure 5b. For a surface source, corresponding to the lowest possible signal, the expected SNR is ∼350 assuming a leakage current of 0.02 pA. A linear relationship was found between the ^220^Rn desorption probability and the detector signal with a *R* ^2^ of 0.996. A high sensitivity of the signal to the desorption probabilities was found with at least 0.5 pA of signal change for every 2 nm change in distribution depth. Therefore, the proposed source strength specification can identify a change in the desorption probabilities due to differing DaRT seed constructions.

**FIGURE 6:**
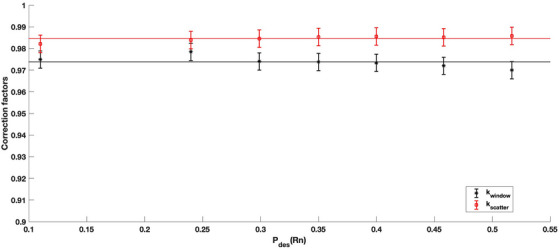
MC‐calculated correction factors as a function of ^220^Rn desorption probability ($P_{des}\left(Rn\right)$). The solid lines indicate the average correction factor values. The error bars correspond to 1σ uncertainty.

Figure 6 displays the MC‐calculated correction factors and their dependence on the investigated range of desorption probabilities. The mean $k_{window}$ correction factor was calculated to be 0.974 with a percent standard deviation of 0.27%. The presence of the mylar window reduces the average energy of the incident alpha particles leading to an increase in stopping power and deposited energy inside the air cavity. Thus, a correction factor of magnitude less than unity is required. A trend in the $k_{window}$ correction factor was noted with the magnitude of the correction increasing with increasing desorption probabilities. The mean $k_{scatter}$ was calculated to be 0.985 with a percent standard deviation of 0.13%. The $k_{scatter}$ correction accounts for both the presence of the vessel walls and the presence of the ion chamber electrodes. Therefore, a correction of less than unity is needed to remove the contribution of both backscattered particles from the collecting electrodes entering the air cavity and the laterally scattered particles into the sensitive volume from the walls of the vessel. The $k_{scatter}$ correction was found to be minimal, to within 2%, with negligible dependence on the desorption probabilities making the correction factor insensitive to the source construction.

## 4 DISCUSSION

To our knowledge, this work is the first to propose an absorbed dose‐base source strength specification for DaRT brachytherapy seeds. It was shown that the ^224^Ra activity as a sole source strength specifier is unsuitable due to significant dependence of absorbed dose to tumor on DaRT seed construction especially the ^224^Ra distribution depth parameter that dictates the progeny radionuclide desorption. Large distribution depths can significantly reduce the ^220^Rn and ^212^ Pb desorption leading to a drastic reduction, of up to 80%, in absorbed dose to tumor over the treatment period. Therefore, measurement of only ^224^Ra activity is insufficient to fully characterize the energy emitted by the source. Variations in progeny desorption behavior are known to influence dose distributions in radionuclide therapies, and further investigation into their impact on spatial dose shifts remains important. The changes in the dose distributions due to varying desorption may require a re‐evaluation of the lattice spacing for the DaRT seed placement pattern.^11^

The proposed absorbed dose standard was found to be highly sensitive to the desorption probabilities allowing a comprehensive measure of DaRT seed strength that includes both ^224^Ra activity and progeny desorption probabilities. Under an assumed ^220^Rn desorption probability of 40%, consistent with values reported in Arazi et al., the system is expected to maintain acceptable SNR for DaRT source activities as low as approximately 0.4 µCi.^19^ Here, an acceptable SNR corresponds to a chamber signal exceeding 1 pA. This sensitivity enables the system to detect small variations in source construction that influences desorption behavior. The ^224^Ra activity is directly proportional to the signal while the relationship between the desorption probabilities and the signal is inverse. Thus, the apparatus was designed to readily measure the ^224^Ra activity using an alpha spectrometer without the removable mylar window so that the ion chamber signal per unit ^224^Ra activity is only dependent on the desorption probabilities.^19^

This approach differs from previous methodologies, such as those by Arazi et al., which utilized a point‐source approximation in MC simulations, whereas our study models the DaRT seed as a distributed surface source.^19^ This distinction is critical, as surface‐based desorption captures spatial distribution effects that are not represented in point‐source geometries. Future comparisons between these approaches may offer insights into differences in dose deposition across tumor volumes. Additionally, simulations by Heger et al. explored lattice configurations for DaRT seeds, examining variations in desorption and diffusion lengths.^11^ While not a direct comparison, their work underscores the importance of modeling desorption behavior accurately to predict dose profiles. Including these considerations in the proposed dosimetry formalism may enhance the accuracy of treatment planning.

The proposed measurement standard has several strengths that contribute to its reliability and reproducibility. First, the use of a continuous flow, vacuum environment and a biased recoil collector ensures the removal of desorbed progeny from the chamber so that only emissions originating from the DaRT source are detected. This design eliminates signal contributions from ^220^Rn decay in surrounding media and minimizes surface contamination. Furthermore, the application of single Coulomb scattering in the MC simulations improved the accuracy of progeny transport modeling, aligning more closely with the physical recoil behavior of alpha‐emitting progeny. Additionally, the MC‐derived correction factors shown in Figure 6 demonstrate the systems robustness to geometric and spectral perturbations. Notably, the trend observed in the $k_{window}$ correction factor, where higher desorption probabilities lead to a larger correction, reflects an increasing spectral contribution from lower energy ^224^Ra emissions (∼5.5–5.7 MeV). These lower energy alphas experience greater relative changes in stopping power upon traversing the mylar window, necessitating a larger correction. The consistency of the $k_{scatter}$ correction across desorption values further supports the measurement systems’ insensitivity to vary source configurations.

Importantly, this system is designed as a primary measurement standard to be implemented at a national metrology institute (NMI), such as NIST, and is not intended for clinical use. The development of a clinically applicable transfer standard instrument is ongoing and remains an area of active investigation. While the current setup is optimized for a 1 cm DaRT seed, the design is easily adaptable. Modifications to the PCB diameter or window aperture would allow for accurate measurements of longer sources, including those up to or exceeding 2 cm in length.

However, there are notable limitations to this study. The surface roughness and inhomogeneities in the actual DaRT source were not explicitly modeled. In reality, the plating of ^224^Ra onto the stainless steel surface may not be uniformly distributed, potentially leading to local variations in progeny desorption that were not captured in this simulation.^45^ Furthermore, the polymer coating typically applied to DaRT sources was not included in the MC model. This exclusion was intentional to isolate the effects of progeny desorption; however, the polymer may introduce attenuation effects that could alter the measured dose distributions. The impact of the polymer layer on recoil containment and surface retention remains a key area for future investigation. Finally, while the current study demonstrates proof‐of‐concept through MC modeling, experimental validation remains necessary to fully confirm the reliability and reproducibility of the proposed absorbed dose standard. A controlled experimental setup with reference DaRT seeds of known desorption behavior would be instrumental in achieving this validation.

The relationship between the ion chamber signal and the desorption probabilities must be empirically determined. Once this relationship is established using multiple reference DaRT seeds with varying measured desorption, obtained from independent alpha spectrometry measurements and MC simulations, the estimated desorption for a given seed can be easily determined and can be used along with the DL model to guide the inter‐seed spacing for a given treatment. Although the proposed absorbed dose standard will reside in a NMI, commercially‐available thin‐wall parallel plate ion chambers can be calibrated against this standard and can be used clinically as transfer standards.

The contribution of alpha particles to the total dose is significantly larger than that of beta particles. However, the potential for alpha particles to be attenuated and lose energy in media other than the tumor, such as the wire or polymer coating of the source, underscores the need for a measurement procedure that accurately characterizes the energy deposition of these therapeutically relevant particles emitted from the DaRT source. The proposed source strength specification is sensitive to these DaRT seed construction parameters and provides a more meaningful measurement. The proposed dosimetry formalism differs from the previously published approach, which used the source as an electrode in a windowless extrapolation chamber geometry.^28^, ^31^ These modifications were necessary due to the inclusion of a window for measuring the DaRT source strength and to perform the experiments in‐vacuo to eliminate progeny radionuclides especially ^220^Rn. Furthermore, the optimal time for measuring the DaRT source strength is approximately six days after seed preparation, when radioactive equilibrium between ^224^Ra and its progeny is achieved. Measuring at this time minimizes variability due to progeny buildup or depletion and ensures a more accurate and reproducible assessment of the source strength.

This work proposes a proof of concept for calibrating the source strength of the DaRT source based on the absorbed dose to air. This represents just one of several effects that must be quantified and standardized to establish a complete dosimetry formalism for the DaRT source. Further work is required to physically measure and standardize the diffusion lengths of ^220^Rn and ^212^ Pb. Validation of the MC model through direct comparison of simulated absorbed dose with experimental measurements using the proposed apparatus will also be necessary. Beyond validating these factors for the dosimetry formalism, additional efforts are needed to characterize relevant transfer instruments that can accurately translate the measurement of absorbed dose to air from alpha and beta emissions of the DaRT source into clinical practice.

## 5 CONCLUSIONS

This work demonstrated the dependence of absorbed dose to tumor on ^220^Rn and ^212^ Pb desorption probabilities and highlighted the need for an absorbed dose‐based source strength specification for DaRT brachytherapy source. A proof‐of‐concept dosimetry protocol using an ion chamber with a thin mylar window was proposed, along with relevant correction factors. The correction factors for the measurements were found to be insensitive to the desorption parameter. The calculated detector signal was found to be highly sensitive to the desorption probabilities allowing an accurate estimation of the dose distribution inside the patient. Ultimately, the proposed measurements aim to improve dosimetry for DaRT treatments.

## CONFLICT OF INTEREST STATEMENT

Larry DeWerd has a financial interest in Standard Imaging Inc.

## Data Availability

Research data are stored in an institutional repository and will be shared upon request with the corresponding author.
